# The Burden of Duodenal Ulcers Among Dyspeptic Patients

**DOI:** 10.7759/cureus.15113

**Published:** 2021-05-19

**Authors:** Abdul Latif, Shahid Karim, Hamid Ali, Manzoor Hussain, Ghulam Mujtaba, Shahid Majid

**Affiliations:** 1 Gastroenterology and Hepatology, Liaquat National Hospital and Medical College, Karachi, PAK; 2 Gastroenterology, Liaquat National Hospital and Medical College, Karachi, PAK; 3 Gastroenterology and Hepatology, The Indus Hospital, Karachi, PAK; 4 Gastroenterology, The Indus Hospital, Karachi, PAK

**Keywords:** dyspepsia, duodenal ulcer, esophagogastroduodenoscopy, smoking

## Abstract

Introduction: Dyspepsia is a common presenting complaint of various upper gastrointestinal disorders. Duodenal ulcer is one of the rare endoscopic findings in patients with dyspepsia, but it can present with upper gastrointestinal bleed. The aim of this study was to determine the frequency of duodenal ulcers among dyspeptic patients undergoing esophagogastroduodenoscopy (EGD).

Subject and methods: All patients who fulfilled the inclusion criteria were recruited during the period of six months, i.e., from July to December 2020 in the Department of Gastroenterology, Liaquat National Hospital and Medical College, Karachi. After obtaining informed and written consent, history, and clinical examination, the EGD was performed to assess the outcome, i.e., frequency of duodenal ulcers.

Results: A total of 156 patients with dyspepsia were included. Eighty-seven (55.8%) were male and 69 (44.2%) were female with the mean age of 36.96+11.71 years. The most common symptom at presentation was epigastric burning seen in 76 patients (48.7%) followed by postprandial fullness in 59 patients (37%). Duodenal ulcers were noted in 18 patients (11.5%) and were significantly associated with alcohol intake, smoking, epigastric pain, postprandial fullness with p-values of 0.001, 0.001, 0.001, and 0.013, respectively.

Conclusion: Duodenal ulcer is an uncommon endoscopic finding in patients with dyspepsia; it is seen in younger age, smokers, alcohol use, and patients presenting with epigastric pain and postprandial fullness.

## Introduction

Dyspepsia is a common gastrointestinal disorder [1] consisting of symptoms such as burning or epigastric discomfort, early satiety, bloating, fullness of the upper belly, or nausea [2]. It is difficult to differentiate between functional and organic causes of dyspepsia on clinical grounds alone which makes it difficult to the evaluation of its etiology. Dyspepsia is one of the most common gastrointestinal complaints in both inpatients and outpatients [3]. An esophagogastroduodenoscopy (EGD) is the investigation of choice for patients with dyspepsia to rule out organic disease [1].

The foremost approach in patients with dyspepsia is recognition of high-risk patients with one or more warning symptoms, especially those over 55 years of age, bleeding, anemia, early satiety, unexplained weight loss, dysphagia, odynophagia, vomiting, family history of gastrointestinal cancer, previous history of esophagogastric malignancy, documented peptic ulcer, upper gastrointestinal surgery, lymphadenopathy, or an abdominal mass [4,5].

There is a substantial number of patients with peptic ulcer diseases including both gastric and duodenal ulcers, seeking surgical opinion worldwide. The concept of acid in peptic ulcer disease, which was the basis for the treatment of peptic ulcer, was revolutionized by the invention of H2 receptor antagonists, which led to the hypothesis of duodenal ulcer acid suppression therapy, that followed decades after the choice of surgical intervention in the form of gastric resections, vagotomies, etc. A triple-drug regimen was established to combat peptic ulcer disease after the discovery of the *Helicobacter pylori* organism as the causative agent and was further altered with concurrent treatment to prevent antibiotic resistance.

Due to a paucity of literature in our population, we designed a study to investigate the prevalence of duodenal ulcers among patients with dyspepsia. This study was conducted to determine the prevalence of duodenal ulcers among dyspeptic patients in our setup. Early recognition and prompt treatment of duodenal ulcers will also help to prevent complications of duodenal ulcers such as bleeding and perforation.

## Materials and methods

This study was conducted after taking approval from Ethical Review Committee (ERC). Patients who visited the Department of Gastroenterology in Liaquat National Hospital, Karachi with dyspepsia, which was defined as chronic or recurrent pain or discomfort centered in the upper abdomen for more than six months, were assessed by history. According to Rome 111 criteria, only one of the four symptoms include postprandial fullness (labeled as positive when unusual fullness after meals), early satiation (labeled as positive when there is an inability to eat a full meal), epigastric pain (labeled as positive when there is epigastric pain for >2 weeks), epigastric burning (labeled as positive when there is epigastric burning for >2 weeks), were enrolled and after taking written and informed consent relevant clinical data were recorded. A brief history regarding demographic variables, risk factors, i.e., alcohol (one drink of alcohol contained 8-10 g of pure alcohol), smoking (five cigarettes per day more than one year), clinical features, and duration of dyspepsia was taken. In all these patients, EGD with Olympus 180 gastroscope system was performed by a consultant gastroenterologist with experience of more than five years. Disruption of duodenal mucosa more than 5 mm with peripheral edema and overlying white exudates was labeled as Forrest's class III ulcer, however, biopsies were not done. The entire data were recorded by a principal investigator on a predesigned performa after reviewing the endoscopic video. Patients of either gender with age between 18 and 60 years having dyspepsia as per operational definition for >6 months who underwent EGD were included, while pregnant women, patients with gastric carcinoma, and chronic liver disease were excluded. Exclusion criteria were followed strictly to avoid the other effect modifiers.

Data analysis

SPSS version 22 (IBM Corp., Armonk, NY) was used for data analysis. Frequencies and percentages were computed for categorical variables like gender, alcohol, smoking (yes/no), clinical features, i.e., epigastric pain, epigastric burning, postprandial fullness, early satiety (yes/no), and duodenal ulcer (yes/no). Quantitative variables were presented as mean ± standard deviation like age, weight height, BMI, and duration of dyspepsia. Effect modifiers like age, BMI, gender, duration of dyspepsia, risk factors (alcohol, smoking), clinical features were controlled through stratification. A Chi-square test was used. P≤0.05 was considered the level of significance.

## Results

A total of 156 patients having dyspepsia were selected to conduct this study. The mean age was 36.967+11.718 years. Eighty-seven patients (55.8%) were males and 69 patients (44.2%) were females. The mean duration of dyspepsia was 14.923+7.777 months. The mean height was 6.971+1.6430 m. The mean weight was 6.971+1.6430 kg. The mean BMI was 6.971+1.6430 kg/m^2^. The descriptive statistics of the age, duration of dyspepsia, height, weight, and BMI is presented in Table 1. The socioeconomic status (monthly income) of patients was <50,000 in 84 patients (53.8%) and >50,000 in 72 patients (46.2%). The risk factors were alcohol (more than one drink per day) in four patients (2.6%) and smoking (five cigarettes per day more than one year) in 14 (9%). The clinical features were epigastric pain in 18 patients (11.5%), epigastric burning in 76 (48.7%), postprandial fullness in 59 (37.8%), and early satiation in 29 (18.6%). In our study, the duodenal ulcers were noted in 18 patients (11.5%), as shown in Table 1.

**Table 1:** Descriptive statistics of age, duration of dyspepsia and BMI and frequency distribution of gender, socioeconomic status, risk factors, clinical features, and duodenal ulcer (n=156)

Variable	Median	IQR		Mean (SD)
Age (years)	35	19		36.9+11.7
Duration of dyspepsia (months)	12	11		11.7+7.7
BMI (kg/m^2^)	24.6	9.5		25.3+5.4
Gender	Frequency (n)		Percentage (%)	
Male	87		55.8%	
Female	69		44.2%	
Total	156		100%	
Socioeconomic status				
<50,000 PKR per month	84		53.8%	
>50,000 PKR per month	72		46.2%	
Total	156		100%	
Risk factors	Yes		Total	
Alcohol (more than one drink per day)	4 (2.6%)		156 (100%)	
Smoking (five cigarettes per day more than one year)	14(9%)		156 (100%)	
Clinical features	Yes		Total	
Epigastric pain	18 (11.5%)		156 (100%)	
Epigastric burning	76 (48.7%)		156 (100%)	
Postprandial fullness	59 (37.8%)		156 (100%)	
Early satiation	29 (18.6%)		156 (100%)	
Duodenal ulcer				
Yes	18		11.5%	
No	138		88.5%	
Total	122		100%	

The frequencies of age groups, gender, duration of dyspepsia, BMI, socioeconomic status, risk factors, clinical features were calculated according to duodenal ulcers. The results are presented in Tables 2-4, respectively. In our study duodenal ulcers were significantly associated with the risk factors (alcohol and tobacco smoking), epigastric pain, and postprandial fullness with the p-value of 0.001, 0.001, 0.001, and 0.013, respectively. 

**Table 2:** Duodenal ulcer according to risk factors (alcohol, smoking; n=156)

	Duodenal ulcer	Total	P-value
	Yes		
Alcohol (more than one drink per day)			
Yes	3 (75%)	4 (100%)	0.001
No	15 (9.9%)	152 (100%)	
Smoking (five cigarettes per day more than one year)			
Yes	14 (100%)	14 (100%)	0.001
No	4 (2.8%)	142 (100%)	

**Table 3:** Duodenal ulcer according to age (years; n=156)

	Duodenal ulcer	Total	P-value
	Yes		
Age (years)			
18-35 years	10 (12.6%)	79 (100%)	0.982
36-60 years	8 (10.4%)	77 (100%)	
Gender			
Male	9 (10.3%)	87 (100%)	0.600
Female	9 (13%)	69 (100%)	
Duration of dyspepsia	Mean (SD)	t-test	P-value
With duodenal	18+11.5	0.72	0.080
Without duodenal	14.5+7.1		
BMI			
With duodenal	23.9+5.8	0.67	0.227
Without duodenal	25.4+5.3		

**Table 4:** Duodenal ulcer according to clinical features (epigastric pain, epigastric burning, postprandial fullness, early satiation)

	Duodenal ulcer	Total	P-value
	Yes		
Epigastric pain			
Yes	16 (88.8%)	18 (100%)	0.001
No	2 (1.5%)	138 (100%)	
Epigastric burning			
Yes	12 (15.8%)	76 (100%)	0.105
No	6 (7.5%)	80 (100%)	
Postprandial fullness			
Yes	2 (3.4%)	59 (100%)	0.013
No	16 (16.5%)	97 (100%)	
Early satiation			
Yes	2 (6.9%)	29 (100%)	0.386
No	16 (12.6%)	127 (100%)	

## Discussion

It is very important for clinicians to consider common causes of dyspepsia in resource-restricted environments, especially where access to the endoscopy service is limited. A negative endoscopy can increase patient comfort and alleviate distress due to fear of severe diseases, considering the poor endoscopy yield in outpatients with dyspepsia [6].^ ^The incidence of duodenal ulcers in our sample was 11.5% compared to the study by Krithika and Mogal [7] in which the prevalence of peptic ulcers was found in 7.5% of the population. In a random population survey of people reporting dyspepsia, Lond et al. [8] observed a frequency of 9% for duodenal ulcers. Katelaris et al. [9]^ ^found a 6% prevalence of duodenal ulcer. In the experiment by Abdeljawad et al. [10], peptic ulcer disease was the most prevalent SEF (4%).

Popular causes of morbidity in Tanzanian patients have been found to be gastritis and peptic ulcer diseases. In addition, the predicted association between duodenal ulcer and *H. pylori* was also shown by this research (patients were given *H. pylori* eradication empirically; no specific tests performed in patients with duodenal ulcer; sign and symptoms improved after *H. pylori* eradication) as documented in other reports from Africa with *H. pylori* infection [11].^ ^There is sufficient evidence regarding the effective role of H. pylori eradication therapy in patients with duodenal ulcers and gastritis [12]. Thus, physicians should test and treat for the infection if resources are available. However, empirical treatment is recommended according to World Gastroenterology Guidelines 2010 in areas with limited resources and unavailability of confirmatory testing.

The risk factors in our study were alcohol (more than one drink a day) in four patients (2.6%) and smoking (five cigarettes per day more than one year) in 14 (9%), in contrast to the Krithika and Mogal [7]^ ^study, where 34% of patients drank beer, 22.5% were smokers, and 19% had a tobacco-chewing habit. In a research by Pertti Aro et al. [13] in Kalixanda study, 17% of patients were smokers. In our study, the clinical features were epigastric pain in 18 patients (11.5%), epigastric burning in 76 (48.7%), postprandial fullness in 59 (37.8%), and early satiation in 29 (18.6%), as compared to Krithika and Mogal [7] study where the majority of patients presented with epigastric pain 83%, followed by retrosternal burning 51%, and acid reflux 49%, each patient presented with three to four symptoms on average. In a thesis done by Locke and Talley [14], the prevalence of heartburn and acid reflux was 42.2% and 45%, respectively. In a series of 309 subjects with dyspepsia by Johnson et al. [15], 125 (40.5%) reported epigastric pain, of which 64 (51.2%) reported no concurrent heartburn, 142 (58%) reported heartburn without abdominal pain, and 61 (24.9%) reported both heartburn and epigastric pain. In our study, the mean duration of dyspepsia was 14.923+7.777 months as compared to Krithika and Mogal [7]^ ^where patients presented with a shorter duration of dyspepsia.

The limitation of this study was a single-center study and smaller size. So further studies with larger sample sizes are required.

## Conclusions

The study concludes that duodenal ulcer is seen only in a small percentage of dyspeptic patients. Duodenal ulcer is more common in younger age, smokers, and alcoholics and in dyspeptic patients who present with epigastric pain or postprandial fullness. It is suggested that policymakers should hold health education campaigns to inform the population regarding the nature of the disease and its risk factors.
